# Ways Community-Based Organizations Enhance Late-Life Depression Care in Partnership With Clinics

**DOI:** 10.1093/geroni/igab046.1710

**Published:** 2021-12-17

**Authors:** Theresa Hoeft, Melissa Gosdin, Jenny Wagner, Stuart Henderson, Mindy Vredevoogd, Katherine James, Jurgen Unutzer, Ladson Hinton

**Affiliations:** 1 University of Washington, Seattle, Washington, United States; 2 University of California Davis, University of California Davis, California, United States; 3 University of Washington, University of Washington, Washington, United States; 4 University of California, Davis, Sacramento, California, United States

## Abstract

Late-life depression is a serious public health concern in the U.S., especially as the population ages. To improve care coordination and increase the number of providers working to improve depression outcomes, primary care clinics and community-based organizations (CBOs) can partner and improve care. Addressing social determinants of health is one area CBOs can help respond to but there are other ways CBOs can bring value to these partnerships with primary care clinics. As part of a qualitative evaluation of the Care Partners Project, 84 key informant interviews and 20 focus groups were conducted over five years with selected primary care physicians, care managers, administrators and psychiatric consultants. These data were coded and organized using an inductive and deductive thematic analysis approach. CBOs contributed to care through 1) adding new services that focus on clients’ social needs (e.g., assistance locating affordable housing, reliable transportation, applying for social security benefits) that were foundational to effective depression care; 2) strengthening core aspects of existing care; 3) incorporating a lay health workforce to enhance care; and/or 4) adding home visits that supported deeper understanding of patient’s life context, enhanced trust and improved access to care. CBOs can enhance depression care through increasing access and quality of care. Findings can inform conversations about the value CBOs offer when partnering with health care systems and improve partnership efforts. Such conversations are worth revisiting as organizations deepen their connections and work together over time.

